# A new approach to transfect NF-κB decoy oligodeoxynucleotides into the periodontal tissue using the ultrasound-microbubble method

**DOI:** 10.1038/ijos.2017.10

**Published:** 2017-04-28

**Authors:** Hiroyuki Yamaguchi, Yuji Ishida, Jun Hosomichi, Jun-ichi Suzuki, Risa Usumi-Fujita, Yasuhiro Shimizu, Sawa Kaneko, Takashi Ono

**Affiliations:** 1 Department of Orthodontic Science, Graduate School of Medical and Dental Sciences, Tokyo Medical and Dental University, Tokyo, Japan; 2 Department of Advanced Clinical Science and Therapeutics, Graduate School of Medicine, The University of Tokyo, Tokyo, Japan

**Keywords:** decoy oligodeoxynucleotide, gene therapy, periodontal tissue, ultrasound

## Abstract

The objective of this study is to investigate the effect of the ultrasound-microbubble technique in nuclear factor kappa B (NF-κB) decoy oligodeoxynucleotide (ODN) transfection in the gingival tissue in mice. The 6-FAM-labeled scrambled decoy ODN with microbubbles was applied to the periodontal tissue in 8-week-old male C57BL/6J mice by ultrasound radiation at low (LUM-Sc) and high (HUM-Sc) intensities to optimize the transfection condition of the ultrasound-microbubble method. Histological inspections were performed two hours after transfection to compare the expression with that in the sham-operated group without ultrasound radiation (A-Sc). Then, an NF-κB decoy was transfected into the periodontal tissue using the high-intensity ultrasound-microbubble (HUM-NF) technique to examine the anti-inflammatory effects of the decoy ODN. Western blot analysis was performed to investigate the expression of interleukin(IL)-1β, IL-6 and intercellular adhesion molecule-1 (ICAM-1) in the gingival tissues in the HUM-Sc, the HUM-NF and control groups. The fluorescence microscopy results showed that the fluorescent intensity in the periodontal tissues in the LUM-Sc and HUM-Sc groups was significantly higher than that in the A-Sc and the control groups. The fluorescent intensity in the HUM-Sc group, especially in the gingival connective tissue, was the highest of all groups. Western blot analysis indicated that the protein expression levels of IL-1β, IL-6 and ICAM-1 in the HUM-NF group were significantly lower than those in the HUM-Sc and the control groups. These findings suggest that the high-intensity ultrasound-microbubble technique is an effective tool for decoy transfection into the periodontal tissue.

## Introduction

Periodontal disease is associated with the infiltration of inflammatory cells into the gingiva, resulting from the interaction between the host’s defence mechanisms and plaque microorganisms^1, 2^ and can lead to the destruction of the periodontal tissues, including alveolar bone loss. Recently, prevention of periodontitis and regeneration of the periodontal tissues has gained increasing attention from many researchers and dental clinicians. Nuclear factor kappa B (NF-κB) is a common signalling molecule involved in inflammation and is known to have an important role in the initiation of immune and inflammatory reactions in periodontal tissues.^3^ Notably, NF-κB was the first transcription factor shown to bind a DNA element in a kappa immunoglobulin light-chain enhancer.^4^ The expression and activation of NF-κB also initiate a downstream signalling cascade involving various inflammatory cytokines, including interleukin(IL)-1β, IL-6 and tumor necrosis factor-α (TNF-α), as well as several adhesion molecules, such as intercellular adhesion molecule-1 (ICAM-1) and vascular cell adhesion molecule-1 (VCAM-1).^5, 6^ In fact, the NF-κB family of transcription factors and their associated signalling pathways have been shown to be involved in both innate and adaptive immune responses.^7^

Notably, due to its substantial role in immunity-related processes, regulation of NF-κB has been utilized as an effective option for treating and/or preventing inflammatory and autoimmune diseases.^8, 9^ In particular, previous studies have demonstrated that NF-κB decoy oligodeoxynucleotide (ODN) transfection is one of the most efficient methods for suppressing NF-κB function in target cells and tissues.^10^ NF-κB decoy ODNs share a sequence similar to that of the NF-κB DNA-binding site and function to selectively block NF-κB activation.^11, 12^ Indeed, several clinical studies have demonstrated that this decoy successfully suppresses the symptoms of various inflammatory and autoimmune diseases, including atopic dermatitis, as well as immunorejection after heart bypass surgery and ischaemic heart disease.^13, 14^

Of particular interest is the methodology used to transfect the decoy ODN into target cells or tissues, which often includes the use of ointments and injections. Recently, a new method using ultrasound technology and microbubbles was proposed for decoy ODN transfection.^15, 16, 17^ A microbubble has a diameter of 1–10 μm, the inside of which is filled with a soluble or sparingly soluble gas.^18^ Microbubbles have the potential to be very proficient drug/gene delivery devices, as they can create small holes in the cell surface, allowing for easy and rapid gene transfection and drug delivery.^19, 20, 21^ Furthermore, Stride *et al.*^22^ developed a novel method for gene transfection that combines microbubble technology and ultrasound radiation. In that study, the core of the cavitation of the microbubble appeared to be altered by ultrasound stimulation, resulting in the production of more holes in the surface of cells close to the microbubble compared with that produced in experiments conducted without ultrasound stimulation. Suzuki *et al.*^23^ and Inagaki *et al.*^24^ also reported the successful transfection of decoy ODNs into arteries using ultrasound-stimulated microbubbles and utilized this technique to investigate the effects of suppressing the expression of certain target genes. Notably, the clinical usefulness and safety of the NF-κB decoy ODN method has been previously demonstrated using percutaneous coronary intervention to prevent arterial restenosis.^14^ However, the ultrasound-microbubble technique has not yet been applied to the transfection of NF-κB decoy ODN into periodontal tissues.

Here, we hypothesized that the ultrasound-microbubble technique could be utilized to effectively transfect the NF-κB decoy through the epithelial layer of gingival tissues. This is the first study in which the ultrasound-microbubble technique was utilized for the delivery of a decoy ODN into periodontal tissues. We first investigated the baseline efficiency of this technique; therefore, we used healthy mice in this study instead of the periodontal disease model mice. The aims of the present study were (i) to apply the ultrasound-microbubble technique to transfer a decoy ODN into the murine periodontium and (ii) to investigate the effects of microbubble-transfected NF-κB decoy ODN on the expression of inflammatory cytokines and adhesion molecules in the gingival tissues of mice.

## Materials and methods

### Animals and decoy ODN transfection

The use and care of all the animals, as well as the experimental procedures utilized in this study, were approved by the Institutional Animal Care and Use Committee and were performed in accordance with the Animal Care Standards of Tokyo Medical and Dental University (#0130359A and #0140298A). Eight-week-old male C57BL/6J mice (*n*=24, CLEA Japan, Tokyo, Japan) were randomly divided into the following four groups: an untreated control group (C), a conventional application group (A-Sc), a low-intensity ultrasound-microbubble group (LUM-Sc) and a high-intensity ultrasound-microbubble group (HUM-Sc; *n*=6 for each group). The mice in the A-Sc group were subjected to transfection of the periodontium with a scrambled decoy ODN conjugated to a fluorescent protein (6-FAM) *via* topical application. In the LUM-Sc and the HUM-Sc groups, after eliminating intraoral water with cotton, transfection of the periodontium with the decoy ODN was carried out *via* the ultrasound-microbubble technique. Notably, ultrasound radiation and microbubble transfection were performed as previously described for arteries.^23, 24^ The gingival tissues of the mice in the LUM-Sc and HUM-Sc groups were irradiated with ultrasound for 60 s at 0.5 W·cm^−2^ (1.0 MHz, duty 50%) and 2.0 W·cm^−2^ (1.0 MHz, duty 50%), respectively.

To investigate the permeability of the decoy ODN into the periodontal tissue after ultrasound-microbubble transfection, we used a 6-FAM-labeled scrambled decoy for fluorescent examination (excitation wavelength, 492 nm; No. 30305603-003; Gene Design, Osaka, Japan). The phosphorothioate scrambled decoy ODN sequences utilized were 5′-TTGCCGTACCTGACTTAGCC-3′ and 3′-GGCTAAGTCAGGTACGGCAA-5′.

**Figure 1:**
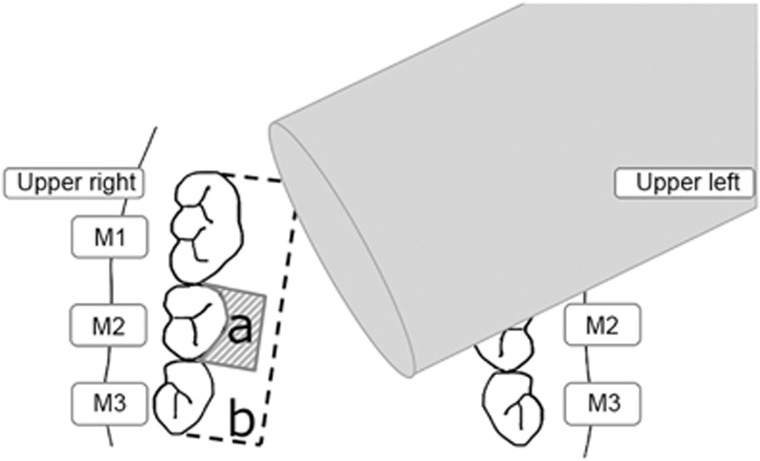
**Schematic illustration of the ultrasound-microbubble method for transfection of palatal gingival tissues**. Immediately after applying the decoy gel to the **a** area (palatal gingival tissue from the first maxillary molar (M1) to the third maxillary molar (M3)), gingival tissues were transfected with the nuclear factor kappa B (NF-κB) decoy oligodeoxynucleotide (ODN) using a Sonitron 2000 device. For western blot analyses, gingival mucosa was collected from the rectangular area (2.5 mm × 2.0 mm) shown in **b** at 2 h post transfection.

For irradiation, we used a Sonitron 2000 ultrasound machine (Nepa Gene, Tokyo, Japan) with a 3.0 mm-diameter probe (Nepa Gene, Tokyo, Japan), according to the manufacturer’s instructions.^25^ A mouth-opening device with a cobalt–chromium alloy wire measuring 0.9 mm in diameter was used to hold the mandibles of mice in the maximum opening position, making the murine oral cavities much more visible during the decoy transfection procedure. As in a previous study, the 6-FAM-labeled scrambled decoy (20 μg in 90 μL) was added to 10 μL of microbubbles (SV-25; Nepa Gene, Tokyo, Japan) and the mixture was suspended in 90 μL of echo gel. All the mice were anesthetized with an intraperitoneal injection of 3.6% chloral hydrate (1 mL per 200 g body weight). The decoy gel was then applied to the right palatal gingiva of the mice in the A-Sc, LUM-Sc and HUM-Sc groups, and the mice in the LUM-Sc and HUM-Sc groups were immediately subjected to ultrasound treatment (Figure 1). All the mice were maintained without any food or water for 2 h after the procedures to ensure the effectiveness of the inoculation. All the mice were killed under excessive anesthesia with chloral hydrate and the maxillae of the mice were dissected and analyzed as described below.

### Quantification of the transfection efficiency of the decoy ODN

Frozen, non-decalcified sections were prepared for histological investigation using a cryofilm transfer kit (Finetec, Gunma, Japan) as previously described.^26, 27^ Isolated maxillae were frozen by quenching in cold hexane, embedded in 5% SCEM gel and again frozen in cold hexane. The frozen SCEM samples were then cut frontally with a disposable carbide tungsten steel blade (Leica Microsystems, Nussloch, Germany). The trimmed surface was covered with an adhesive film (Finetec, Gunma, Japan), and each sample was sectioned frontally along with the film at a thickness of 10 μm. For histological analysis of the periodontal tissue, the slices were stained with hematoxylin and eosin (Leica Microsystems, Nussloch, Germany) and observed with an optical microscope (ECLIPSE 80i; Nikon, Tokyo, Japan).

Fluorescence images were obtained using a confocal laser-scanning microscope (FV10i-DOC; Olympus, Tokyo, Japan). In total, five slides from the serial frontal sections of the upper right second maxillary molar in each mouse were selected for analysis. We focused on three defined regions of interest (50 μm × 50 μm) in each section: the gingival epithelium, the gingival connective tissue and the alveolar bone. The fluorescence intensities of the 6-FAM-positive cells in these regions were calculated using FLUOVIEW software (Olympus, Tokyo, Japan), and the fluorescence intensity was calculated three times for each section; these measurements were then averaged for each sample. Furthermore, the average greyscale values of all pixels from each of the five slides analyzed was calculated to obtain the mean fluorescence intensity value for each mouse.^28^

### NF-κB decoy ODN transfection

In the previous experiment, the decoy transfection performed on the HUM-Sc group was the most effective. Therefore, the same protocol used on the HUM-Sc group in the first experiment was used for our investigation of the downstream effects of NF-κB suppression on the expression of inflammatory cytokines in murine periodontal tissues. For this subsequent experiment, 8-week-old male C57BL/6J mice (*n*=18, CLEA Japan) were randomly divided into the following three groups: a control group, the scrambled decoy experimental group (HUM-Sc) and the NF-κB decoy experimental group (HUM-NF; *n*=6 for each group). The NF-κB decoy ODN and the scrambled decoy ODN were transfected into the HUM-NF and HUM-Sc groups, respectively, using the same ultrasound-microbubble technique described for the HUM-Sc group in the experiment outlined above. The phosphorothioate NF-κB decoy ODN sequences utilized were as follows: 5′-CCTTGAAGGGATTTCCCTCC-3′ and 3′-GGAGGGAAATCCCTTCAAGG-5′.

### Western blot analysis

Western blot analysis was utilized to evaluate the expression of IL-1β, IL-6 and ICAM-1 in mouse gingival tissues. Briefly, palatal gingival tissues were collected (2.0 mm × 2.5 mm) from mice in all the three groups 2 h after transfection. Total protein was then extracted from each tissue using RIPA buffer (Thermo Fisher Scientific, Waltham, MA, USA) and soluble proteins were resolved via 10% sodium dodecyl sulfate-polyacrylamide gel electrophoresis and transferred to a polyvinylidene fluoride membrane (Immobilon-P, Millipore, Darmstadt, Germany). The blots were subsequently probed with a primary antibody for IL-6 (1:1 000; Novus Biologicals, Littleton, CO, USA), IL-1β (1:1 000; Santa Cruz Biotechnology, Dallas, TX, USA) or ICAM-1 (1:1 000; Proteintech, Chicago, IL, USA). Equal protein loading was confirmed by probing for β-actin(1:2 000; Santa Cruz Biotechnology). For chemiluminescence detection, we used a peroxidase-conjugated anti-rabbit IgG secondary antibody (1:10 000; Santa Cruz Biotechnology). The band intensities were measured using a ChemiDoc image analysis system (Fuji Film, Tokyo, Japan).

### Statistical analysis

After testing for normality and equal variances, intergroup comparisons were conducted *via* one-way analysis of variance and Tukey’s *post hoc* testing. The results are presented as the means±standard errors (*n*=6 mice for each). Differences were considered to be significant at *P*<0.05.

## Results

### Transfection of decoy ODN into periodontal tissue *via* an ultrasound-microbubble method

**Figure 2:**
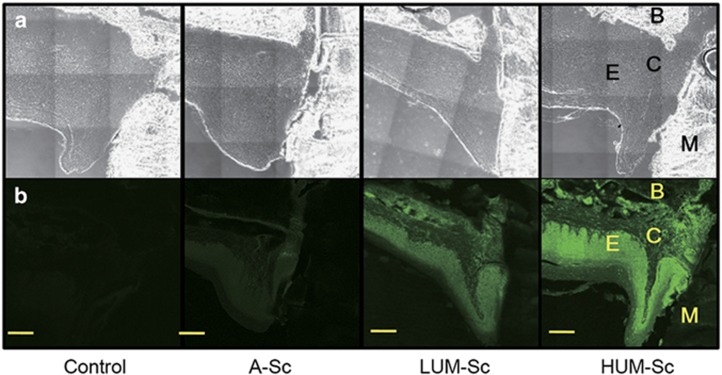
**Histological images of mouse periodontal tissues after transfection of nuclear factor kappa B (NF-κB) decoy oligodeoxynucleotides (ODNs)**. The right maxillary second molar (M2) was sectioned into 10 μm-thick frontal slices. The scrambled decoy penetration was examined by confocal fluorescence microscopy in the control, conventional application (A-Sc), low-intensity ultrasound-microbubble (LUM-Sc) and high-intensity ultrasound-microbubble (HUM-Sc) groups. (**a**) Phase-contrast images and (**b**) fluorescence images (432 nm). B, alveolar bone; C, gingival connective tissue; E, gingival epithelium; M, maxillary right second molar. Scale bar=50 μm.

**Figure 3:**
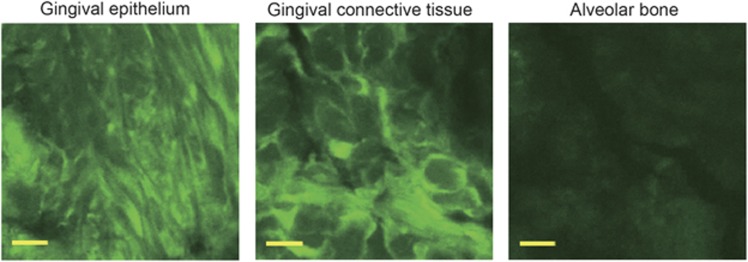
**Magnified views of periodontal tissue in the high-intensity ultrasound-microbubble (HUM-Sc) group**. High magnification of the gingival epithelium (E), gingival connective tissue (C), and alveolar bone (B) in Figure 2. Scale bar=10 μm.

**Figure 4:**
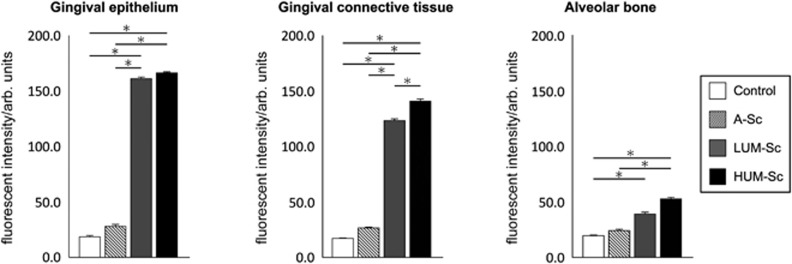
**Quantification of fluorescence intensity in 6-FAM-positive areas**. The intensity of 6-FAM fluorescence within the areas outlined in Figure 2 (50 μm × 50 μm) was evaluated by measuring the densitometry of the fluorescence in each image. The results are expressed as the means±standard errors (*n*=6) in arbitrary (arb.) units. *P<0.05. A-Sc, scrambled decoy in control conventional application (without ultrasound); HUM-Sc, scrambled decoy using high-intensity ultrasound-microbubble; LUM-Sc, scrambled decoy using low-intensity ultrasound-microbubble.

First, we examined the feasibility of transfecting a decoy ODN into murine periodontal tissue using a previously established ultrasound-microbubble method. Following transfection, the maxillae samples were sectioned and subjected to hematoxylin-and-eosin-staining analysis. Notably, there were little-to-no differences in the periodontal tissue structure, infiltration of inflammatory cells or blood vessel numbers between groups (data not shown), indicating that the ultrasound radiation did not damage the gingival tissue in any way. Furthermore, we compared the transfection efficiency of the decoy ODN by analyzing the levels of 6-FAM-labeled scrambled decoy ODN fluorescence between groups. Fluorescence was clearly detected in the periodontal tissues of both the HUM-Sc and LUM-Sc groups, while no expression was detected in the untreated control group (Figure 2). Meanwhile, there were only a few 6-FAM-positive cells in the A-Sc group. High magnification images of the periodontal tissues showed that the intense fluorescent signal observed in the HUM-Sc tissues was localized to the surface area of the alveolar bone. Moreover, both the gingival connective tissues and the gingival epithelia of the mice in this group were 6-FAM-positive (Figure 3). In fact, while there was no significant difference in the fluorescence intensity between the control and A-Sc groups in the alveolar bone, gingival connective tissue or gingival epithelium, there was a significant difference in intensity between the control group and both of the ultrasound groups, LUM-Sc and HUM-Sc (Figure 4). A difference in fluorescent intensity was also observed between the LUM-Sc and HUM-Sc groups, particularly in the gingival connective tissue, with that of the HUM-Sc group being greater (Figure 4). Together, these data indicate that the following conditions, as used for the HUM-Sc group in these experiments, comprise an optimal method for decoy ODN delivery into gingival tissues: ultrasound irradiation for 60 s at a frequency of 1.0 MHz, an intensity of 2.0 W·cm^−2^ and a duty cycle of 50%.

### Suppression of inflammatory protein production in gingival tissues following NF-κB decoy transfection

**Figure 5:**
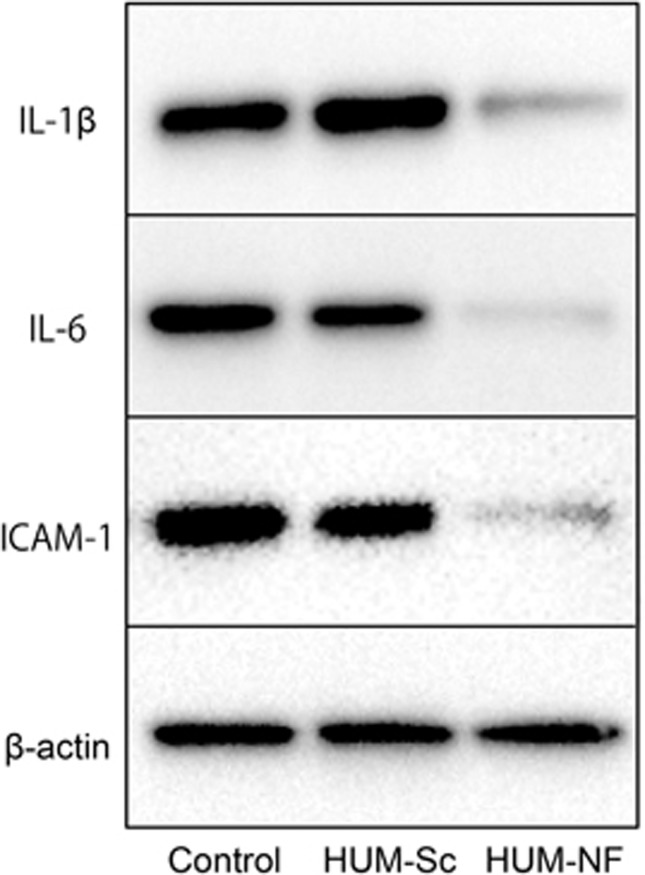
**Western blot analysis of IL-6, IL-1β and ICAM-1 expression in gingival tissues**. Representative images of western blot analysis of IL-6, IL-1β and ICAM-1 expression in palatal gingival tissue lysates collected 2 h after transfection with the nuclear factor kappa B (NF-κB) decoy oligodeoxynucleotide (ODN) via the ultrasound-microbubble method. β-Actin served as a loading control. ICAM-1, intercellular adhesion molecule-1; IL, interleukin; HUM-NF, NF decoy using high-intensity ultrasound-microbubble; HUM-Sc, scrambled decoy using high-intensity ultrasound-microbubble.

**Figure 6:**
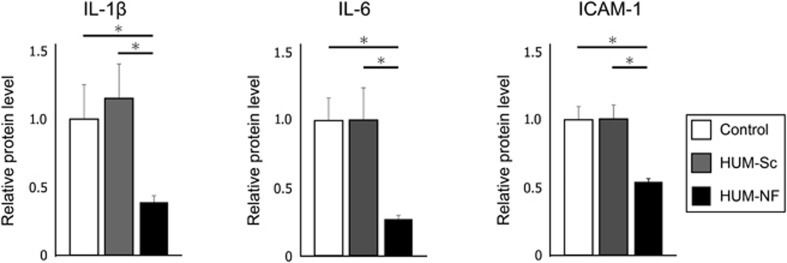
**Quantification of IL-6, IL-1β and ICAM-1 protein levels in gingival tissues.** The protein expression levels of IL-6, IL-1β and ICAM-1 were measured by densitometry analysis of the western blot images presented in Figure 5. The band intensities were normalized to that of β-actin, and the expression level of each protein was compared with that in the control group. The results are expressed as the means±standard errors (*n*=6) of relative protein levels. *P<0.05. ICAM-1, intercellular adhesion molecule-1; IL, interleukin; HUM-NF, nuclear factor decoy using high-intensity ultrasound-microbubble; HUM-Sc, scrambled decoy using high-intensity ultrasound-microbubble.

To investigate the effects of the decoy ODN on NF-κB expression, the mice were treated with the NF-κB-specific decoy using the optimized protocol outlined above. Western blot analysis showed that the protein expression levels of IL-1β, IL-6 and ICAM-1 in the control group were comparable to those in the HUM-Sc group, which was transfected with the scrambled decoy (Figure 5). Conversely, the mice in the HUM-NF group exhibited significantly lower levels of these inflammatory proteins than those in the control and HUM-Sc groups (Figure 6).

## Discussion

Many new drug/gene delivery methods have been developed to treat gingival tissues, including antimicrobial drug-eluting implants,^29^ the delivery of naked plasmid DNA with ultrasound and bubble liposomes,^30^ and decoy ODN transfection via tissue injection.^31^ In this study, we first sought to determine the feasibility of using the ultrasound-microbubble technique, which was developed by Inagaki *et al.*^24^ as a means to administer medications to injured arterial tissues and thereby suppress neointima formation, for transfecting decoy ODNs into gingival tissues. Notably, this method has been shown to enable noninvasive transfection of decoy ODNs, genes or drugs in an efficient, rapid and focused manner. In the present study, microbubble treatment resulted in the formation of small holes within cell membranes, which would have allowed the immediate passage of genes, drugs or in this case, the decoy ODN, into the cell cytoplasm,^21^ that then disappeared after a short period, with no lasting damage.^32^ In an oral environment, it is essential that medication be delivered quickly within a short period because saliva makes it difficult to maintain the contact of drugs with the target area and to ensure an effective density of the therapeutic agents. The results of this study indicate that the ultrasound-microbubble technique can be successfully used to transfect a decoy ODN immediately into gingival tissues, suggesting that this methodology should be evaluated for its efficacy in delivering other genes and drugs to oral and other tissues.

Although the ultrasound-microbubble method is feasible in this cellular context, determining the optimal ultrasound conditions for our system was also necessary. To do so, we investigated the transfection efficiency of a 6-FAM-labelled scrambled decoy into periodontal tissues in the presence of low (LUM-Sc) or high intensity (HUM-Sc) compared with that in the non-ultrasound-microbubble-transfected (A-Sc) and the untreated control groups. In particular, we chose to perform these experiments on healthy gingival tissues of mice instead of on periodontal lesions to exclude the potential influence of inflammatory conditions on the transfection efficiency. Our data demonstrate that the fluorescent intensity of both the HUM-Sc and LUM-Sc gingival epithelial tissues was higher than that of the two groups that did not undergo ultrasound treatment. Notably, while the epithelial tissues of the LUM-Sc and the HUM-Sc groups exhibited similar fluorescence intensities, the fluorescence intensity of the gingival connective tissues in the HUM-Sc group was markedly higher than that in the LUM-Sc group. Therefore, it would appear that the conditions utilized for the HUM-Sc group resulted in improved decoy transfection, especially in the connective tissue of the gingiva, suggesting that not only are these particular conditions optimal for decoy transfection into gingival tissues but that there may be a relationship between the power of the ultrasound radiation and the infiltration of the decoy ODN. However, additional work is necessary to fully explore this phenomenon.

In the second stage of our study, we utilized the optimized HUM-Sc conditions to transfect an NF-κB decoy ODN into the gingiva of mice and thereby investigate the effects of NF-κB suppression in this tissue. In the current literature, NF-κB is widely accepted to be the key regulator of inflammatory cytokines and cell adhesion molecules during the immune response.^33^ Typically, NF-κB is bound to its inhibitor and is retained in the cell cytoplasm. However, when stimulated by mechanical stress, cytokines, free radicals or endotoxins, such as lipopolysaccharide, NF-κB is activated *via* the phosphorylation of its inhibitor. The activated NF-κB then translocates into the nucleus, where it regulates the expression of numerous genes.^34^ For instance, monocyte-derived inflammatory cytokines such as TNF-α and IL-1β have been demonstrated to be regulated by active NF-κB and to, in turn, increase the expression of ICAM-1 and VCAM-1 *via* the NF-κB signalling pathway.^35^ Notably, the NF-κB decoy ODN has also been previously utilized as a suppresser of inflammatory cytokines and adhesion molecules during various types of inflammatory diseases, including cardiovascular disease,^24, 36^ atopic dermatitis,^37^ periodontitis^31^ and others.^38, 39, 40^ Moreover, the NF-κB decoy ODN was recently used in clinical studies as a component in a new ointment for atopic dermatitis^13, 41^ and as a novel treatment that could prevent coronary restenosis.^42^ In a previous study, Shimizu *et al.*^31^ observed inhibition of alveolar bone resorption and suppression of inflammatory cytokine expression within canine periodontal lesions injected with the NF-κB decoy ODN as a treatment for periodontitis. However, given the invasive nature of this method, the development of a simple, effective and noninvasive approach for the administration of the decoy into periodontal lesions is highly desired. In this study, we therefore evaluated the efficacy of a noninvasive method comprising the combination of ultrasound and microbubbles for transfection of the NF-κB decoy ODN into target gingival tissues.

To evaluate the downstream effects of decoy ODN-mediated NF-κB suppression in gingival tissues, we examined the expression of IL-1β, IL-6 and ICAM-1. Notably, all the cytokines and cell adhesion molecules investigated are known to have a role in the progression of periodontal disease. In periodontal bone metabolism, IL-1β is known to stimulate bone resorption, inhibit bone formation and induce the production of matrix metalloproteinases and procollagenases, which promote the destruction of periodontal structures and tissues.^43^ In addition, Stashenko *et al.*^44^ suggested that there is a positive correlation between IL-1β expression in the gingiva and the level of attachment loss. Meanwhile, the primary function of IL-6, a cytokine involved in the immune response, in periodontal tissues is the induction of final B-cell maturation into immunoglobulin-secreting plasma cells.^45^ IL-6 has also been suggested to potentially act as an autocrine and/or paracrine factor in bone resorption in pathological states by stimulating the formation of osteoclasts and activating osteoclastic bone resorption.^46^ Finally, the cell adhesion molecule ICAM-1 is a transmembrane protein that is often found in endothelial tissues and leukocyte cells and has been shown to have a role in cell–cell interactions. Furthermore, ICAM-1 appears to facilitate the endothelial transmigration of leukocytes in the initial stage of gingival inflammation.^47, 48, 49^ As these essential proteins are all directly regulated by NF-κB, this transcription factor is thought to be critical during periodontitis.

In our analysis, the tissues in the LUM-Sc group exhibited lower expression of IL-1β, IL-6 and ICAM-1 than that observed in the HUM-Sc and the control groups. In contrast, there was no significant difference in cytokine expression between the HUM-Sc and the control groups. Interestingly, previous studies have shown that ultrasound techniques can cause heat damage on target tissues.^50, 51^ However, our results illustrate that ultrasound radiation alone did not affect cytokine expression or tissue histology in the gingival regions subjected to ultrasound treatment. Thus, it would appear that the ultrasound-microbubble method is a useful technique for transfecting the decoy ODN into gingival tissues without severe side effects. Indeed, using this approach, we successfully suppressed the function of NF-κB, which, in turn, resulted in the suppression of several downstream signalling molecules. The function of the NF-κB decoy as a competitive antagonist of NF-κB activation to inhibit the overexpression of inflammatory cytokines and cell adherence molecules is a crucial distinction from that of corticosteroids, which inhibit inflammatory and immune reactions. Indeed, clinical administration of an NF-κB decoy to patients with atopic dermatitis resulted in no major adverse effects on the immune response.^52, 53^ On the other hand, western blot analyses showed that the NF-κB decoy ODN suppressed the protein expression of IL-1β, IL-6 and ICAM-1 in healthy gingival tissues. These findings indicate that we cannot exclude the possibility of immunosuppression by the transfection of the NF-κB decoy using the ultrasound-microbubble technique. Therefore, further studies are necessary.

As this study is, to the best of our knowledge, the first to investigate the feasibility of the ultrasound-microbubble technique for periodontal tissue treatment, further studies are needed to better understand gingival inflammation and disease. For example, while the effects of NF-κB transfection into healthy gingival tissue were the main focus of this study, the effects of such transfection on periodontal lesions and diseased tissue should be examined using mouse models of periodontal inflammation, bacterial infection or systemic inflammation to elucidate the potential applications for this methodology of drug delivery in clinical cases of periodontal disease. In addition, in the current study, we analyzed the transfection efficiency and protein expression levels of immune-related proteins within the gingiva at only 2 h post transfection. As such, further research is required to characterize the pharmacokinetics of the decoy ODN within periodontal tissues over a longer period. Meanwhile, a previous study demonstrated that NF-κB decoy ODN transfection accelerated the regeneration of the alveolar bone loss region.^31^ Therefore, it would also be interesting to determine how this ultrasound-microbubble approach could be applied to mouse models of alveolar bone deficiency to further investigate the effects of NF-κB on alveolar bone regeneration.

## Conclusions

In the present study, we demonstrated that transfection of the NF-κB decoy using the ultrasound-microbubble technique resulted in decreased expression of IL-1β, IL-6 and ICAM-1 within the periodontal tissues of mice. These findings suggest that the ultrasound-microbubble technique is an effective method for decoy transfection within this particular cellular context and should therefore be evaluated for the introduction of other genes, decoy ODNs and drugs into oral tissues. Our method may also have therapeutic potential for periodontal disease, particularly during the initiation and healing stages, without the need for invasive measures or severe side effects. However, additional studies are needed to investigate the clinical application of ultrasound-microbubble decoy ODN transfection for the treatment of other kinds of inflammatory diseases.
